# Process-oriented analysis of dominant sources of uncertainty in the land carbon sink

**DOI:** 10.1038/s41467-022-32416-8

**Published:** 2022-08-15

**Authors:** Michael O’Sullivan, Pierre Friedlingstein, Stephen Sitch, Peter Anthoni, Almut Arneth, Vivek K. Arora, Vladislav Bastrikov, Christine Delire, Daniel S. Goll, Atul Jain, Etsushi Kato, Daniel Kennedy, Jürgen Knauer, Sebastian Lienert, Danica Lombardozzi, Patrick C. McGuire, Joe R. Melton, Julia E. M. S. Nabel, Julia Pongratz, Benjamin Poulter, Roland Séférian, Hanqin Tian, Nicolas Vuichard, Anthony P. Walker, Wenping Yuan, Xu Yue, Sönke Zaehle

**Affiliations:** 1 College of Engineering, Mathematics and Physical Sciences, University of Exeter, Exeter, EX4 4QF UK; 2 Laboratoire de Météorologie Dynamique, Institut Pierre-Simon Laplace, CNRS-ENS-UPMC-X, Paris, France; 3 College of Life and Environmental Sciences, University of Exeter, Exeter, EX4 4RJ UK; 4 Karlsruhe Institute of Technology, Institute of Meteorology and Climate Research/Atmospheric Environmental Research, 82467 Garmisch-Partenkirchen, Germany; 5 Canadian Centre for Climate Modelling and Analysis, Climate Research Division, Environment and Climate Change Canada, Victoria, BC Canada; 6 Laboratoire des Sciences du Climat et de l’Environnement, LSCE/IPSL, CEA-CNRS-UVSQ, Université Paris-Saclay, F-91198 Gif-sur-Yvette, France; 7 CNRM, Université de Toulouse, Météo-France, CNRS, Toulouse, France; 8 Department of Atmospheric Sciences, University of Illinois, Urbana, IL 61821 USA; 9 Institute of Applied Energy (IAE), Minato-ku, Tokyo 105-0003 Japan; 10 National Center for Atmospheric Research, Climate and Global Dynamics, Terrestrial Sciences Section, Boulder, CO 80305 USA; 11 Hawkesbury Institute for the Environment, Western Sydney University, Penrith, NSW Australia; 12 CSIRO Oceans and Atmosphere, Canberra, ACT 2101 Australia; 13 Climate and Environmental Physics, Physics Institute and Oeschger Centre for Climate Change Research, University of Bern, Bern, Switzerland; 14 Department of Meteorology, University of Reading, Reading, UK; 15 Max Planck Institute for Meteorology, Bundesstr. 53, 20146 Hamburg, Germany; 16 Max Planck Institute for Biogeochemistry, Jena, Germany; 17 Ludwig-Maximilians-Universität München, Luisenstr. 37, 80333 München, Germany; 18 NASA Goddard Space Flight Center, Biospheric Sciences Laboratory, Greenbelt, MD 20771 USA; 19 Schiller Institute for Integrated Science and Society, Department of Earth and Environmental Sciences, Boston College, Chestnut Hill, MA 02467 USA; 20 Climate Change Science Institute & Environmental Sciences Division, Oak Ridge National Lab, Oak Ridge, TN 37831 USA; 21 School of Atmospheric Sciences, Sun Yat-sen University, Zhuhai, Guangdong 510245 China; 22 School of Environmental Science and Engineering, Nanjing University of Information Science and Technology (NUIST), Nanjing, China

**Keywords:** Carbon cycle, Carbon cycle, Attribution

## Abstract

The observed global net land carbon sink is captured by current land models. All models agree that atmospheric CO_2_ and nitrogen deposition driven gains in carbon stocks are partially offset by climate and land-use and land-cover change (LULCC) losses. However, there is a lack of consensus in the partitioning of the sink between vegetation and soil, where models do not even agree on the direction of change in carbon stocks over the past 60 years. This uncertainty is driven by plant productivity, allocation, and turnover response to atmospheric CO_2_ (and to a smaller extent to LULCC), and the response of soil to LULCC (and to a lesser extent climate). Overall, differences in turnover explain ~70% of model spread in both vegetation and soil carbon changes. Further analysis of internal plant and soil (individual pools) cycling is needed to reduce uncertainty in the controlling processes behind the global land carbon sink.

## Abstract (web summary)

The global net land sink is relatively well constrained. However, the responsible drivers and above/below-ground partitioning are highly uncertain. Model issues regarding turnover of individual plant and soil components are responsible.

## Introduction

Over the last 60 years, there has been a continuous rise in anthropogenic CO_2_ emissions. Around the equivalent of a quarter of these emissions have been taken up by the land biosphere (known as the natural land sink), acting as a strong negative feedback to mitigate climate change^1^. To be able to project the global carbon cycle (and hence the climate response) in the future, we need to understand the underlying processes and their timescales that drive the contemporary land sink. There are many distinct but interdependent mechanisms that regulate the flow of carbon into, through, and out of the land. The timescales at which these processes (e.g. photosynthesis, allocation, plant growth, litterfall, plant mortality and soil turnover) act on the carbon cycle range from days to centuries, and the interplay between these determines the changes in land carbon storage^2^.

Recent work suggests the global net land sink is located in northern latitudes^3^. The attribution of drivers is highly uncertain, but a combination of increasing atmospheric CO_2_ concentrations^4^, reactive nitrogen (N) deposition^5^ and atmospheric warming^6^ are likely responsible. Tropical lands are probably nearer net zero carbon sinks due to large land-use and land cover changes (LULCC) carbon losses counteracting the ‘natural’ sink^7,8^. Atmospheric observations also show that the Amazon forest is currently carbon neutral (‘natural’ sink = LULCC-driven source)^9^, and this provides further evidence of a limited net tropical sink. Yet, the largest gross fluxes between the land and atmosphere are often found in tropical regions^10^, and so changes in tropical ecosystem functioning can have significant global impacts.

Process-based dynamic global vegetation models (DGVMs) that simulate processes of carbon uptake and release can help to elucidate the roles of individual drivers (rising atmospheric CO_2_, changes in climate, nutrient deposition, and LULCC), attribute the change to processes, and quantify regional sinks over timescales needed given the multitude of timescales over which terrestrial processes act (i.e. beyond the period of reliable empirical and remote-sensed data).

DGVMs are used to estimate the natural land sink (hereafter simply land sink) as part of the global carbon budget (GCB^8^). Globally, the DGVM multi-model mean estimate of the global land sink is consistent with the global carbon budget residual land sink (the difference between fossil and land-use emissions and atmospheric and ocean sinks, see Table 5 in ref. 8), however, there is a significant spread across models, and here we show the spread widens at regional scales, when quantifying changes in vegetation and soil carbon, or attributing changes in internal processes to external drivers. Therefore, while model ensembles are helpful in analysing global-scale processes, they must be interpreted in the context of their process representation and unique and collective biases^2,4^.

These deficiencies are of high significance when trying to understand past changes but also when DGVMs are used to make predictions of future carbon cycling^11^, as limited trust can be placed in projections when certain fundamental processes and carbon-climate feedbacks are not fully captured^2^. Therefore, it is of high priority to identify the leading sources of uncertainty in DGVMs and understand the relationship between drivers and processes on varying spatial scales. In this study, we use a triple (3D-matrix) approach to identify where models agree and, just as importantly, disagree, and thus guide future modelling efforts:

- What are the (1) external drivers (concurrent rises in atmospheric CO_2_ and N deposition, climate and LULCC), (2) main regions (tropics and extra tropics) and (3) processes (production vs turnover) primarily responsible for the changes in the global net land carbon sink?

We use the suite of 18 DGVMs from the GCB2021 (TRENDYv10; ref. 8) to quantify changes in net carbon exchange and carbon stocks over the period 1959–2020. TRENDYv10 provides a set of simulations to attribute these changes to drivers, and we use a process attribution framework to decompose changes in carbon stocks into those driven by productivity and turnover separately. This framework enables us to express productivity and turnover-induced changes in carbon stocks in units of *PgC*, which allows for a direct comparison between both processes, which have units of carbon per unit time and time, respectively (see Methods).

## Results

### Drivers of global and regional land sinks

**Fig. 1:**
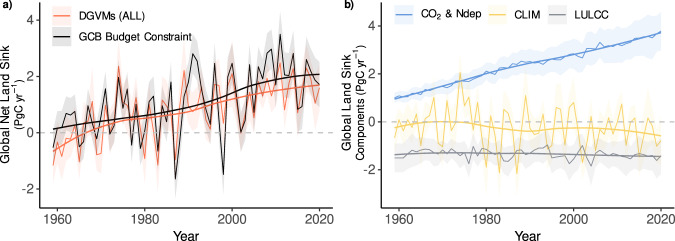
Global net land sink and attribution to drivers. **a** Net annual land carbon sink (PgC yr^−1^) as estimated by dynamic global vegetation models (DGVMs) with all drivers varying (red) and the top-down Global Carbon Budget (GCB) constraint (black) and **b** the decomposition of the DGVM net sink into contributions from rising atmospheric CO_2_ concentrations and N deposition (blue), changes in climate (yellow), and land-use and land cover change (grey). Thick lines show a locally fitted regression. Shading around DGVM estimates corresponds to 1σ, and the uncertainty on the GCB constraint is taken from ref. 8.

**Fig. 2:**
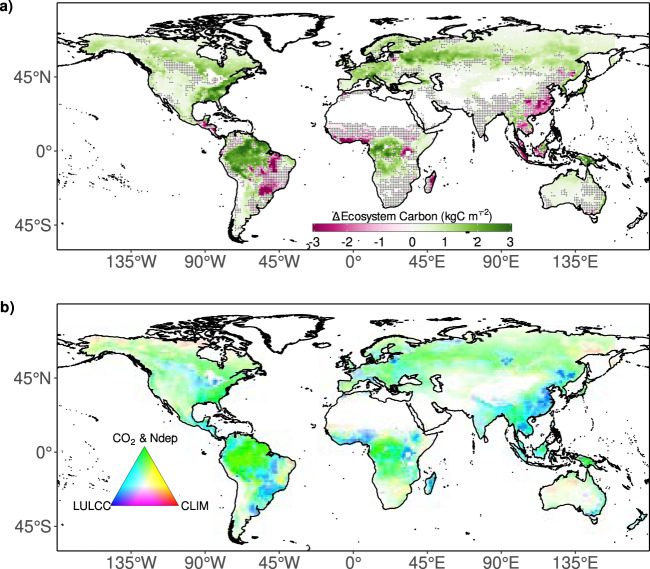
Driver attribution to spatial changes in total ecosystem carbon. Maps show the multi-model mean **a** net change in ecosystem (vegetation and soil) carbon (kgC m^−2^) from 1959–2020 and **b** the contribution of each driver to overall change; CO_2_ and N deposition (green), climate (red), and land-use and land cover change (blue). Stippling in panel **a** indicates <80% of models agree on the direction of change. The colours in panel **b** are calculated by assigning a red-green-blue (RGB) value to each grid depending on the relative magnitude of change due to each driver. Transparency is determined by the magnitude of the net change in panel **a**.

The global net land sink is derived from the difference between the fossil fuel emissions (E_FOS_) and the CO_2_ accumulation in the atmosphere (G_ATM_) and uptake by the oceans (S_OCEAN_). We refer to this estimate of the net land sink as the ‘observed’ *GCB* budget constraint (= E_FOS_-G_ATM_-S_OCEAN_; see methods and ref. 8) which grew from 0.2 ± 0.4 (mean ± std. dev) PgC yr^−1^ in the 1960s to 1.7 ± 0.6 PgC yr^−1^ in the decade 2011–2020, with DGVMs (S3 simulation; see Methods) capturing the increase (−0.1 ± 0.6 to 1.6 ± 0.5 PgC yr^−1^; Fig. 1). DGVMs suggest the enhanced net sink over the past 60 years has mainly been driven by rising atmospheric CO_2_ concentrations and nitrogen deposition (1.2 ± 0.2 PgC yr-1 over 1960–1969 rising to 3.5 ± 0.8 PgC yr-1 over 2011–2020) (Fig. 1b), with the sink partially offset by relatively constant net LULCC emissions of 1.3 ± 0.5 PgC yr^−1^ (predominantly arising from tropical deforestation and shifting cultivation^12^; Fig. 2 and Supplementary Fig. 1). DGVMs indicate that climatic variability drives the large year-to-year changes (±2 PgC yr^−1^) in the land sink (Fig. 1), with long-term (multi-decadal) climate trends reducing the land sink since the 1980s (−0.4 ± 0.5 PgC yr^−1^ over 1980–2020) (Fig. 1b).

The CO_2_ and N deposition-driven land carbon sink is located in northern and tropical forests (Fig. 2). LULCC-induced carbon losses occur across the globe but are most apparent in tropical latitudes and certain hotspot regions across the northern hemisphere (China, USA and West Eurasia), which recently have been (within our study period) densely forested (Fig. 2 and Supplementary Fig. 1). There are signs of European carbon sink in part driven by a/reforestation or natural land (re)establishment. The impact of changes in climate can also be detected across the globe, with vegetation and soil carbon losses in the Amazon, the Sahel and South Africa, and climate-induced carbon gains in east Brazil, Australia, and across the high northern latitudes (Fig. 2 and Supplementary Fig. 1). In summary, the DGVM ensemble agrees on the large sink located in the world’s forests. However, the models are not in full agreement in the direction of change in ecosystem carbon across much of the globe, in particular in regions with competing CO_2_ and LULCC effects (Fig. 2a).

### Key processes and uncertainties behind the sink

The top-down global GCB budget constraint combined with the bottom-up DGVM estimates give high confidence the land has been a net sink of carbon over the last 60 years, however, the relative contribution of each driver (e.g. CO_2_, N deposition, climate, LULCC), and ecological attribution (vegetation, soil) of the sink remains elusive.

**Fig. 3:**
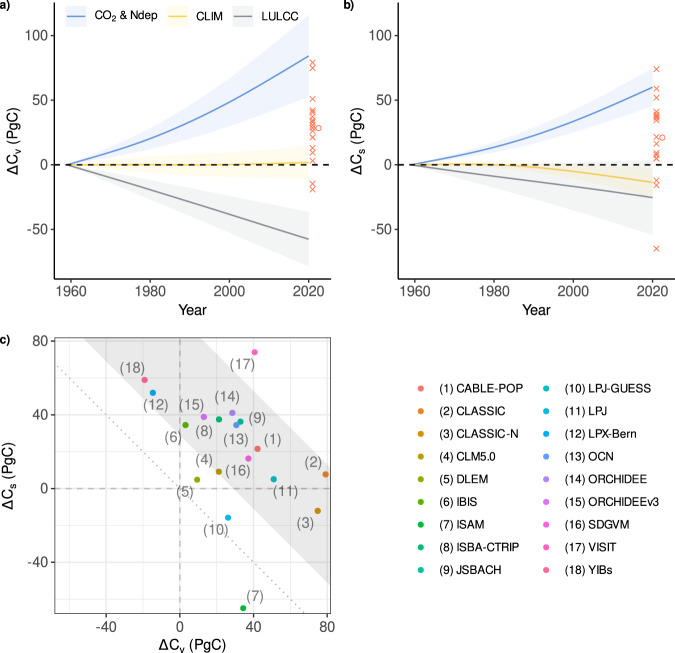
Temporal changes in global vegetation and soil carbon stocks. Time-series show the change in global **a** vegetation (${\varDelta C}_{v}$) and **b** soil (${\varDelta C}_{s}$) carbon stocks from 1959–2020 due to each of the three external drivers (CO_2_ and N deposition, climate, land-use, and land cover change). Lines represent the mean of the dynamic global vegetation models (DGVMs) and shading the ±1σ of the DGVMs. The DGVM output is first smoothed using a fourth-order spline. The cumulative net (sum of three drivers) change in global carbon stocks by 2020 is shown (red crosses show each model and red circle shows the model mean). **c** Shows the change in vegetation and soil stocks for each of the 18 models and the grey region is the Global Carbon Budget net land sink constraint (see Methods).

The DGVM multi-model mean suggests a net global increase in both vegetation (*ΔC*_*v*_ = 28 ± 26 (mean ± std. dev) PgC) and soil (*ΔC*_*s*_ = 21 ± 32 PgC) carbon stocks, although the uncertainty is large (Fig. 3a, b), with two models (LPX-Bern and YIBs) simulating a net loss of vegetation carbon and three models (CLASSIC-N, ISAM and LPJ-GUESS) simulating a net loss of soil carbon (Fig. 3c).

This relatively high uncertainty is in part due to large opposing fluxes (Fig. 3a) driven by increasing atmospheric CO_2_ and N deposition (carbon increase) vs LULCC (carbon decrease at global scale), with soil carbon gains (from rising CO_2_) also counteracted by negative fluxes driven by climate change (Fig. 3b). In general, there are no observational constraints on long-term (pre-satellite era) changes in global vegetation or soil carbon, and so reducing model uncertainty is challenging. Therefore, although the DGVMs generally capture the global net carbon sink, the attribution to vegetation or soil stocks is completely unconstrained (Fig. 3c) and is driven by alternate model structures and parameterisations between DGVMs.

DGVMs have inherently different baseline productivity, allocation, and turnover rates as well as varying degrees of sensitivity of processes to environmental change. To gain a deeper understanding of the processes driving carbon sink changes, we use our attribution framework (see Methods) to associate the modelled changes in vegetation and soil carbon to changes in inputs (NPP for vegetation and litterfall for soil) and outputs (turnover rates for vegetation and soil). For each of these processes, we identify their changes due to CO_2_, climate change or LULCC, according to the models.

**Fig. 4:**
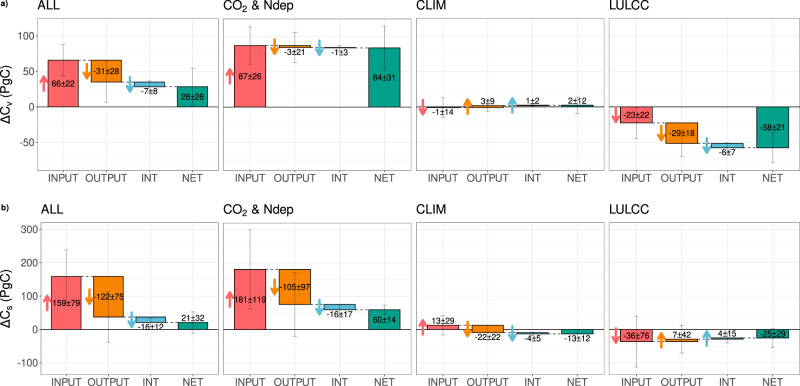
Process and driver attribution of changes in global vegetation and soil carbon stocks. Change in global **a** vegetation (${\varDelta C}_{v}$) and **b** soil (${\varDelta C}_{s}$) carbon stocks over 1959–2020 (PgC). The contribution to net changes in carbon stocks (green bars) from changes in *inputs* (net primary productivity for vegetation ($\varDelta {{{{{{\mathrm{NPP}}}}}}}{\tau }_{v,1959}$) and vegetation to soil flux for soil ($\varDelta {f}_{{vs}}{\tau }_{s,1959}$), red bars), outputs/turnover (${{{{{{\mathrm{NP}}}}}}}{{{{{{\mathrm{P}}}}}}}_{1959}\varDelta {\tau }_{v}$ for vegetation and ${f}_{{vs},1959}\varDelta {\tau }_{s}$ for soil, orange bars), and the interaction term ($\varDelta {{{{{{\mathrm{NPP}}}}}}}\varDelta {\tau }_{v}$ for vegetation and $\varDelta {f}_{{vs}}\varDelta {\tau }_{s}$ for soil, blue bars) are shown. The bars depict the multi-model mean with the range as ±1σ of the models. The arrows show the direction of change in carbon stocks due to each process. The panels from left to right show the changes due to all drivers varying (ALL), changes in atmospheric CO_2_ and N deposition, climate (CLIM), and land-use and land cover change (LULCC).

The ensemble-simulated increase in global vegetation carbon over the last 60 years (*ΔC*_*v*_ = 28 ± 26 PgC; Fig. 4a) was driven by enhanced plant productivity in response to rising CO_2_ concentrations and N deposition, with small (but highly uncertain) losses due to changes in vegetation turnover ($\varDelta {\hat{C}}_{v,{{{{{{\mathrm{input}}}}}}},{{{{{{\mathrm{CO}}}}}}}2}$ = 87 ± 26 PgC, $\varDelta {\hat{C}}_{v,{{{{{{\mathrm{output}}}}}}},{{{{{{\mathrm{CO}}}}}}}2}$ = −3 ± 21 PgC; Fig. 4a). Although the central estimate of the net CO_2_ and nitrogen deposition biomass response appears relatively well constrained at global scale ($\varDelta {C}_{v,{{{{{{\mathrm{CO}}}}}}}2}$ = 84 ± 31 PgC), model estimates range from a gain of 30 PgC (YIBs) to a gain of 150 PgC (LPJ) in biomass due to rising atmospheric CO_2_ (Supplementary Fig. 2).

**Fig. 5:**
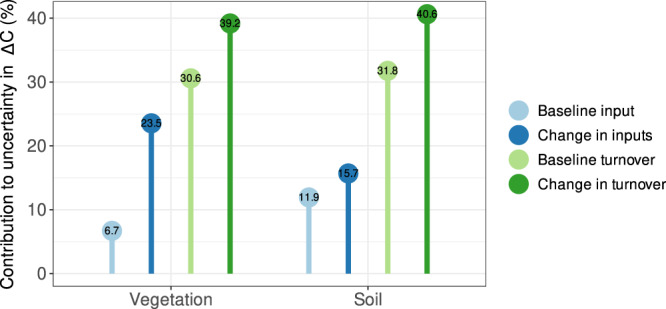
Attribution of uncertainty to processes in modelled changes in carbon stocks. The relative uncertainty (defined as the standard deviation among model estimates) in the change in global vegetation ($\varDelta {C}_{v}$) and soil ($\varDelta {C}_{s}$) carbon stocks resulting from each of the driving terms (Eqs. 9 and 10 in Methods). The four terms are baseline input (baseline productivity ${{{{{{{\mathrm{NPP}}}}}}}}_{1959}$) for vegetation and baseline litterfall/mortality (${f}_{{vs},1959}$) for soil), change in inputs (change in productivity $\varDelta {{{{{{\mathrm{NPP}}}}}}}$) for vegetation and change in litterfall/mortality ($\varDelta {f}_{{vs}}$) for soil), baseline turnover (${\tau }_{v,1959}$ for vegetation and ${\tau }_{s,1959}$ for soil), and change in turnover ($\varDelta {\tau }_{v}$ for vegetation and $\varDelta {\tau }_{s}$ for soil). For each of these terms in Eqs. 9 and 10, we calculate the standard deviation in $\varDelta {C}_{v}$and $\varDelta {C}_{s}$using the multi-model mean values of all other terms in the equations and the individual model values for that term.

Model spread is driven by a combination of uncertainty in the response of both NPP (e.g. driven by uncertainty in leaf-level photosynthetic response, scaling to canopy and landscape scales, carbon allocation and nutrient limitation) and vegetation turnover (*τ*_*v*_) (e.g. driven by the treatment of nutrient limitation coupled with differences in allocation and in some cases forest-stand packing constraints^13^) to rising atmospheric CO_2_ and N deposition (Fig. 5 and Supplementary Fig. 3). There is some confidence in the direction of change in global and regional NPP, albeit not in the magnitude, whereas models disagree on the direction of *Δτ*_*v*_ due to rising CO_2_ (Fig. 4). CO_2_ and N deposition-driven *Δτ*_*v*_ depends on changes in stand dynamics (in some models) and plant allocation. For example, an increase in short-lived root production, at the expense of wood growth, to alleviate nutrient limitations on plant growth can reduce *τ*_*v*_. This increase in root allocation also changes vegetation nitrogen demand (because of the substantially higher C:N in wood compared to roots), which feeds back onto the whole-plant productivity response to rising CO_2_. Conversely, some models increase their wood allocation fraction when NPP (or production) increases, which will increase *τ*_*v*_^14^.

This increase in vegetation stocks has been partially offset by emissions associated with LULCC from vegetation ($\varDelta {C}_{v,{{{{{{\mathrm{LULCC}}}}}}}}$ = −58 ± 21 PgC; Fig. 4a), predominantly in the tropics (Supplementary Fig. 1), which increases vegetation turnover (loss of biomass to the atmosphere or wood products). About half ($\varDelta {\hat{C}}_{v,{{{{{{\mathrm{input}}}}}}},{{{{{{\mathrm{LULCC}}}}}}}}$ = −23 ± 22 PgC, ~40%; Fig. 4a) of the net LULCC-driven global vegetation carbon losses are from a reduction in inputs (NPP) to the land, as crops or pastures often have lower productivity than the forests that they replace. The other half ($\varDelta {\hat{C}}_{v,{{{{{{\mathrm{output}}}}}}},{{{{{{\mathrm{LULCC}}}}}}}}$ = −29 ± 18 PgC; Fig. 4a) is attributable to changes in turnover related to forests containing woody biomass, which has a slower turnover than leaves and fine roots. The turnover effect is further aggravated through the phenomenon that due to their longer-lived biomass, forests take up more carbon under rising CO2 levels, but this sink is lost by clearing for agricultural use. This is known as the “loss of additional sink capacity” (LASC)^15,16^ and accounts for the impact of environmental (CO_2_ and climate) changes on carbon uptake of deforested land (found in S3 simulation) compared to potential vegetation (found in S2 simulation). Ref. 16 attributes ~40% of DGVM estimated LULCC vegetation and soil carbon losses to the LASC, albeit with a different methodology and study period.

All models simulate a net loss of global biomass following LULCC, although the magnitude is not well constrained. Model spread is driven by differences in LULCC process representation in models^7^. We find that models that include wood harvest (routine harvest of established managed forests), grazing harvest, or shifting cultivation simulate larger *C*_*v*_ losses than models without these land management processes (Supplementary Fig. 4), in line with earlier studies showing the high importance of land management on vegetation carbon stocks^17^. Further, in northern ecosystems, models do not agree on the direction of $\varDelta {{{{{{\mathrm{NPP}}}}}}}$ or *Δτ*_*v*_ following LULCC (Supplementary Fig.  5a). In addition to the land management processes mentioned above, uncertainty in large-scale regional changes in *C*_*v*_ is in part driven by smaller-scale regional compensations, where losses in Russia and USA are somewhat countered by a forest regrowth sink in Eurasia (Supplementary Fig. 1). Therefore, the balance between these two opposing fluxes determines net northern changes in *C*_*v*_ from LULCC, with uncertainties in both carbon loss fluxes and regrowth uptake^18,19^. In addition, spatial patterns of northern LULCC losses and gains are not entirely consistent between models (Supplementary Fig. 6), which adds to uncertainty in local, regional, and hemispheric net LULCC-driven changes in vegetation carbon.

Changes in climate have a much smaller but similarly uncertain influence on vegetation processes at a global scale, with no agreement among models on the direction of change in biomass driven by shifts in NPP ($\varDelta {\hat{C}}_{v,{{{{{{\mathrm{input}}}}}}},{{{{{{\mathrm{CLIM}}}}}}}}$ = −1 ± 14 PgC over 1959–2020) or vegetation turnover ($\varDelta {\hat{C}}_{v,{{{{{{\mathrm{output}}}}}}},{{{{{{\mathrm{CLIM}}}}}}}}$ = 3 ± 9 PgC) at global scale (Fig. 4a). However, models align closer at regional scales, where northern warming has stimulated productivity ($\varDelta {\hat{C}}_{v,{{{{{{\mathrm{input}}}}}}},{{{{{{\mathrm{CLIM}}}}}}},{{{{{{\mathrm{North}}}}}}}}$ = 11 ± 6 PgC; Supplementary Fig. 5a), and in the tropics, reductions in productivity (due to warming and/or changes in precipitation) lead to losses of vegetation ($\varDelta {\hat{C}}_{v,{{{{{{\mathrm{input}}}}}}},{{{{{{\mathrm{CLIM}}}}}}},{{{{{{\mathrm{Tropics}}}}}}}}$ = −12 ± 12 PgC; Supplementary Fig. 5b). Overall, these opposing regional trends cancel and explain the small global signal and the model uncertainty at the global scale.

Increased production and loss of biomass enhance soil inputs ($\varDelta {\hat{C}}_{s,{{{{{{\mathrm{input}}}}}}},{{{{{{\mathrm{ALL}}}}}}}}$ PgC) and drives the growth in global soil carbon stocks (*ΔC*_*s*_ PgC), albeit with low confidence in the magnitude (Fig. 4b). Our analysis also indicates that enhanced CO_2_ concentrations increase soil turnover, which is a direct result of enhanced inputs to the fast-turnover litter and surface soil pools. Our methodology simplifies the TRENDY model structures into a single soil pool to represent the entire soil system, and so a relative increase in the *faster* pools moves the aggregate soil pool turnover (*τ*_*s*_) towards that of the litter and surface soil pools—a phenomenon known as false-priming^20^. In addition, changes to soil conditions (e.g. increased soil moisture^13^) following increased atmospheric CO_2_ can impact *τ*_*s*_, although it is difficult to separate the false-priming and actual changes in *τ*_*S*_ with the set of simulations used in this study. This simplification leads to low confidence in the partition of net soil carbon changes into the input and turnover-driven changes, whereas the net change is well constrained ($\varDelta {C}_{s,{{{{{{\mathrm{CO}}}}}}}2}$PgC).

Reduced litterfall (input to soil) due to the conversion of forests to agricultural land is the predominant pathway of soil carbon loss, in line with previous studies^21^ ($\varDelta {\hat{C}}_{s,{{{{{{\mathrm{input}}}}}}},{{{{{{\mathrm{LULCC}}}}}}}}$ = −36 ± 76 PgC, $\varDelta {C}_{s,{{{{{{\mathrm{LULCC}}}}}}}}$ = −25 ± 29 PgC). This is particularly evident in the tropics (Supplementary Fig. 7b) with large-scale deforestation over the last several decades, while deforestation and reforestation are more in balance in the extra tropics. However, there is relatively high uncertainty in the magnitude of LULCC impacts on soil carbon inputs ($\varDelta {\hat{C}}_{s,{{{{{{\mathrm{input}}}}}}},{{{{{{\mathrm{LULCC}}}}}}}}$) (Fig. 4b), with models ranging from losses of over 100 PgC to gains of 45 PgC over the period 1959–2020. Land management processes such as crop and wood harvesting (in particular, the frequency of harvest and fraction of biomass removed and respired elsewhere) are leading uncertainties in simulating LULCC impacts on soil carbon (Supplementary Fig. 4)^22,23^. Turnover-driven changes play less of a role in soil C changes in our models. This could be due to offsetting effects such as a decrease in soil C due to the altered quality of the litter input (a higher fraction of faster decomposing material for agriculture as compared to forests) or a reduction in fire-related losses with the transformation of natural ecosystems^21^.

Increased heterotrophic respiration rates and subsequent reductions in the turnover time of the soil pool due to global warming entirely offset the increase in soil carbon from climate-enhanced NPP and litterfall (Fig. 4b). Overall, changes in climate caused a net loss ($\varDelta {C}_{s,{{{{{{\mathrm{CLIM}}}}}}}}$ = −13 ± 12 PgC) of global soil carbon. We find the impact of climate change on carbon storage is accelerating, in particular for northern soil and tropical biomass, where carbon stock changes from increased/decreased productivity (for northern soil and tropical biomass, respectively) have accelerated by 19 TgC yr^−2^ and 9 TgC yr^−2^, since the turn of this century (Supplementary Fig. 8). This equates to annual mean carbon gain/losses due to changes in productivity of 1.6 PgC yr^−1^ and −0.3 PgC yr^−1^ over 2001–2020 compared to 0.6 PgC yr^−1^ and −0.2 PgC yr^−1^ over 1961–2000, for northern soil and tropical biomass, respectively (Supplementary Fig. 8b, e). This acceleration is likely driven by warming in the north (2001–2020 April-August mean 0.9 °C warmer than 1961–2000) and the tropics (2001–2020 annual mean 0.6 °C warmer than 1961–2000)^24,25^, as well as potential changes in sensitivity of the biosphere to warming^26^.

## Discussion

The ‘observed’ GCB global net land sink is captured by the TRENDYv10 DGVM ensemble. We find a robust agreement that rising atmospheric CO_2_ concentrations and N deposition drive the land carbon sink, and changes in climate along with LULCC lead to a source of carbon in the atmosphere. Further, the DGVMs corroborate that the net land sink is located in northern latitudes, with the tropics more carbon neutral, in line with recent independent top-down estimates^3,8^.

However, the models do not entirely agree on the partition of the net land sink between vegetation and soil. Thirteen of 18 models indicate an increase in global vegetation and soil carbon stocks, although the magnitude of change is highly unconstrained (ranging from ~0 to +80 PgC over the past 60 years). The remaining five models simulate a reduction in either vegetation or soil stocks, highlighting the disparity between models regarding internal carbon cycling processes.

There are no observations of global carbon stocks covering the entire study period, but a recent synthesis of observation-based biomass change estimates in global forests suggests a net biomass sink of 0.3–2.1 PgC yr^−1^ since 2000^27^, giving confidence to the models that simulate net gains. However, these observational studies do not constrain the magnitude of the vegetation sink any more than the DGVM mean biomass sink of −0.1 to 1.7 PgC yr^−1^ (averaged over 2001–2020). Furthermore, we cannot even be certain in the direction of change in global soil carbon as direct observations of large-scale changes in soil carbon stocks do not exist^2,4^.

Our analysis indicates that baseline vegetation turnover rates and the turnover response to rising atmospheric CO_2_ and N deposition are the key uncertainties in modelled *ΔC*_*v*_, with a smaller but still significant contribution from changes in NPP. Differences in modelled vegetation turnover are driven by variations in whole-plant mortality rates, simulated tissue lifespan, and the allocation of NPP to plant components with inherently different turnover rates and their responses to changing environmental conditions^14,28^. For example, some models may reduce plant mortality and turnover as atmospheric CO_2_ concentrations rise. Increased carbon stores could supply maintenance respiration in periods of photosynthetic stress^29,30^, and increases in water-use efficiency may alleviate the impact of drought^31,32^. In contrast, shifts in plant community composition or self-thinning dynamics may increase stand-level mortality^33^. These structural differences lead to no agreement on the direction of *Δτ*_*v*_ in the models presented here.

In addition to turnover-related uncertainties, the increase in NPP and biomass due to rising CO_2_ is not well constrained. The choice of photosynthesis and stomatal model determines the leaf-level response, with a wide range of implementations and outcomes in DGVMs^34^. Scaling from leaf to canopy level causes further discrepancies due to different structural assumptions between models as to the vertical distribution of light, nitrogen, photosynthetic capacity, and the treatment of sunlit and shaded leaves^34^. Further, NPP and biomass production is constrained by stoichiometric nutrient requirements. Nitrogen availability mediates the biomass response to rising CO_2_^35^, and therefore it is likely that differences in the inclusion and representation of carbon-nitrogen coupling between models contribute to uncertainty in *ΔC*_*v*_^36,37^. Overall, these structural uncertainties manifest as a four-fold range in estimates of the CO_2_ effect on NPP and biomass (gains of 41 PgC (CLASSIC-N) up to 157 PgC (ISAM) over the previous 60 years).

Furthermore, LULCC-driven reductions in NPP and woody carbon stocks have removed a substantial portion of global vegetation carbon. However, the exact magnitude of carbon loss is not well constrained by the DGVMs. Biases in the carbon density of deforested land^38^ and model-specific choices of whether to replace forest or grassland for agricultural expansion are leading sources of error when calculating LULCC losses^39^. Our results highlight that, firstly, variation in the representation of relevant processes, in particular, forest management (e.g. treatment of wood harvest) and shifting cultivation, leads to systematic differences between modelled *ΔC*_*v*_^7,40^. Second, DGVMs are missing carbon losses associated with degraded forests, which can exceed deforestation losses, indicating simulated LULCC losses could be underestimated^40–42^. Finally, uncertainty exists with LULCC maps used to drive DGVMs as historical land-use is not perfectly known and products differ in the land cover types and transitions included^40^.

The models suggest that soil has been a net carbon sink globally over the past six decades, although there is over 100% uncertainty on the magnitude (21 ± 32 PgC). This relatively large range is due to the opposing impacts of rising CO_2_ and climate/LULCC effects which partly cancel out. Further, the impact of LULCC on soil carbon is also difficult to quantify robustly with the suite of DGVMs used here, as models do not agree on the sign of $\varDelta {f}_{{vs}}$ or $\varDelta {\tau }_{s}$ following LULCC. In general, changes in soil carbon stocks are difficult to constrain due to a lack of large-scale observations. It is likely historical LULCC caused additional carbon fluxes as a result of land erosion^43,44^, degradation of agricultural soils^45,46^, and losses from drained peatlands^47^. These management processes are not included in models and most do not simulate peatlands at all, potentially causing errors in simulated soil carbon fluxes.

Overall, the soil carbon sink is caused by increased litter inputs from CO_2_-driven vegetation growth. There is some evidence additional litter and root exudates enhance soil carbon stocks when nitrogen availability is high^48^. It is important to stress that the model simulations include widespread nitrogen deposition, which may have helped to sustain a strong CO2 response of biomass and soil carbon sinks^5,49^.

However, additional soil inputs can accelerate organic matter decomposition to release plant-available nitrogen (via priming effects^50^), which reduces soil carbon storage^51–53^. DGVMs do not simulate priming effects or explicitly account for microbial activity, mineral association, aggregation, or mycorrhizal fungi interactions, all of which regulate soil carbon turnover and accumulation rates^54^. DGVMs represent soil carbon with a set of cascading pools, where decomposition losses are determined by first-order decay rates (although this is an active area of model development^55^). Therefore, the modelled soil sink has to be interpreted in the context of model structure and the limited ability of DGVMs to capture governing soil processes.

In general, it is difficult to attribute processes (soil inputs vs turnover) to changes in soil carbon for two main reasons. First, soil model structure leads to false-priming effects^20^. The apparent reduction in soil turnover is a consequence of increased surface soil carbon relative to deep soil, as opposed to a change in actual turnover rates. Second, our process attribution methodology treats the soil as a single pool. Therefore, the calculation of ${\tau }_{s}$ ($\frac{{C}_{s}}{{R}_{h}}$) may include deep, inactive (on decadal timescales) soil carbon, and underestimate the turnover rate of the active soil. Hence, our process attribution of soil carbon changes is not well constrained. For example, we estimate a 300 PgC increase in soil carbon from increased inputs for CLM5.0, a result which is in part driven by the large carbon stocks in high northern latitudes leading to a large estimate of ${\tau }_{s}$^56^. We do account for this bias by scaling output to actual changes in modelled carbon stocks (see methodology), but our results highlight the urgent need for evaluation of individual carbon pools and fluxes (leaf, root, wood and each soil pool) separately, and not bulk terms.

In addition to the CO_2_-driven sink, evidence exists for a substantial impact of forest regrowth on the northern sink over the previous 60 years^18,19^. DGVMs have some capacity to capture regrowth effects (predominantly following land abandonment), however, most do not simulate forest demography or detailed forest management (e.g. enhanced stock densities^57^) and so could underestimate the actual regrowth carbon sink^58^. Further, not all DGVMs simulate disturbance in unmanaged land (e.g. wind and pests), and therefore, natural disturbances beyond fire and the subsequent regrowth will not be captured by the DGVMs here^18^. Moreover, not all models simulate fire mortality, and fire models do not always capture observed spatiotemporal patterns and associated carbon emissions^59^, as well as large uncertainties due to issues with forcing data^60^.

In addition to direct human-caused carbon losses, alternative drivers of mortality can reduce carbon stocks. For example, climate-induced mortality, combined with a shorter tree lifespan resulting from faster growth^61,62^, currently weaken the Amazon forest sink^63^. However, DGVMs show no sign of increased carbon turnover due to changes in climate or faster growth, although this discrepancy is not unexpected as DGVMs do not include detailed mortality processes, e.g. drought-mortality^64^, although this is an area of current model development^65,66^. The DGVMs do indicate a decline in tropical productivity due to climate change, in line with remote-sensing and upscaled in-situ observations^67^, indicating current temperatures may exceed tropical plant thresholds^24^.

Here we have shown that the long-term evolution of the global net land sink is well estimated by an ensemble of state-of-the-art DGVMs. However, a complete process-based understanding is still lacking due to several model shortcomings. Specifically, above/below-ground partitioning of the sink is highly uncertain due to too simplistic representation of internal carbon cycling. Model improvement into plant allocation, tissue lifespan and mortality, as well as the inclusion of process-based soil carbon and nutrient cycling, should be a high priority moving forward.

## Methods

### TRENDYv10 models

We analyse output from 18 DGVMs that are part of a recent model intercomparison project, TRENDYv10 and the Global Carbon Budget 2021 (GCB2021)^8^. The models included in the analysis here are CABLE-POP, CLASSIC, CLASSIC-N, CLM5.0, DLEM, IBIS, ISAM, ISBA-CTRIP, JSBACH, LPJ-GUESS, LPJ, LPX-Bern, OCN, ORCHIDEE, ORCHIDEEv3, SDGVM, VISIT, and YIBs (see ref. 8 for model descriptions and setup). One model (JULES-ES-1.1) from TRENDYv10 is not included due to incomplete data. Note, CLASSIC-N^68^ and ORCHIDEE^69^ were not part of GCB2021 due to the inclusion of alternate model versions (CLASSIC and ORCHIDEEv3) but are included in TRENDYv10 and this study.

The models are forced with a merged monthly Climate Research Unit (CRU)^70^ and 6-hourly Japanese 55-year Reanalysis (JRA-55)^71^ data set. The models are also forced with atmospheric CO_2_^72^, gridded nitrogen deposition^73^ and nitrogen fertiliser^74^. DGVMs use the HYDE (v3.3) land-use change data set^75,76^, which provides annual pasture and cropland areas at a global scale, and includes improvements in the spatial distribution of agricultural regions^77^. Several models (CABLE-POP, CLM5.0, JSBACH, LPJ-GUESS, LPJ, and VISIT) also use harmonised land-use change data (LUH2-GCB2021^8^), which provides information on sub-grid-scale land-use transitions.

To isolate the response of the land to each driver (CO_2_, climate, LULCC), each model performs four simulations: S0 (fixed pre-industrial atmospheric CO_2_ and land-use, recycled 1901–1920 climate), S1 (transient atmospheric CO_2_, recycled 1901–1920 climate, and fixed pre-industrial land-use), S2 (transient atmospheric CO_2_, transient climate, and fixed pre-industrial land-use), and S3 (transient atmospheric CO_2_, transient climate, and transient industrial land-use). Nitrogen deposition varies temporally in simulations S1–S3. Therefore, the transient CO_2_ (+N deposition) effect on the ‘natural’ land sink is calculated by S1−S0, S2−S1+S0 is the climate effect on the ‘natural’ land sink, S3−S2 is the LULCC effect, and S3 is the net effect. There exists an artefact in the HYDE3.3 data causing a large land-use transition and emission peak around 1960. To correct this, we replace the LULCC estimates for 1959–1961 with the average of 1958 and 1962 in each DGVM.

Net Biome Productivity (NBP) from the S3 simulation represents the net land sink and can therefore be compared to the ‘observed’ Global Carbon Budget land constraint, which is calculated in the GCB as fossil fuel emissions—atmospheric carbon growth rate—ocean carbon sink (see Fig. 1 in the main text and Table 5 in ref. 8 and data taken from 10.18160/gcp-2021). Atmospheric CO_2_ concentration measurements began in 1959^78^, and so this independent constraint on the land sink covers the period 1959–2020, which defines the study period used throughout our analysis.

### Data processing

First, we calculate for each model the global and regional (two regions: north of 30°N and south of 30°N) annual mean values for NBP, net primary productivity (NPP), heterotrophic respiration (${R}_{h}$), vegetation carbon (${C}_{v}$) and soil carbon (${C}_{s}$), which includes litter (${C}_{{{{{{{\mathrm{litter}}}}}}}}$) and coarse woody debris (${C}_{{{{{{{\mathrm{CWD}}}}}}}}$) for 1959–2020. We exclude the product pool from our analysis as we do not have carbon emission data from wood products available.

### Process attribution framework

To attribute changes in C pools to different processes, we quantify C pool changes attributable to changes in productivity and changes in turnover time. We do this using an analytical approximation of C pool dynamics corrected for non-steady state and more complicated behaviour of the models. First, we approximate the steady-state C pool size given the inputs and turnover time of a given pool in a given year. We then quantify the change in the steady-state pool sizes estimated for 1959 and the year in question and partition this change proportionally among inputs and turnover time. Finally, to adjust to non-steady state conditions and more complicated model structures (i.e. multiple pool dynamics, not a single pool as in this approximation), we adjust the attributed proportions by adding the difference in actual modelled change in carbon stock and the steady-state approximation, scaled by the relative magnitude of productivity and turnover-driven changes.

The change in carbon pools can be expressed as:

1 $$\frac{{dC}}{{dt}}={{{{{{\mathrm{INPUT}}}}}}}-{{{{{{\mathrm{OUTPUT}}}}}}},$$

where we define $\frac{{dC}}{{dt}}$ as the change in carbon pool per year. To quantify the internal processes impacting the output flux, we can define ${{{{{{\mathrm{OUTPUT}}}}}}}=\frac{C}{\tau }$, where $\tau$ is the mean turnover time (the inverse of the mean turnover rate) of the carbon pool, C. Therefore, we can rewrite Eq. 1 as:

2 $$\frac{{dC}}{{dt}}={{{{{{\mathrm{INPUT}}}}}}}-\frac{C}{\tau }$$

We separate the land into two main pools, vegetation (${C}_{v}$) and soil (${C}_{s}$). ${C}_{v}$ is a direct model output and includes leaf, root and wood biomass. Similarly, ${C}_{s}$,${C}_{{{{{{{\mathrm{litter}}}}}}}},{C}_{{{{{{{\mathrm{CWD}}}}}}}}$ are direct model outputs, which we sum together for our analysis. The rest of the methodology, ${C}_{s}$refers to the sum of these three pools. Not all models simulate litter and CWD pools. ISAM does not simulate litter and only CABLE-POP, CLM5.0, DLEM and LPJ-GUESS simulate CWD. Further, for CLM5.0, the litter pool is incorporated in the soil pool output, and so is also excluded from the sum to avoid double counting.

We can now specify Eq. 2 for vegetation and soil as:

3 $$\frac{d{C}_{v}}{{dt}}\;=\;{{{{{{\mathrm{NPP}}}}}}}-\frac{{C}_{v}}{{\tau }_{v}}$$

4 $$\frac{d{C}_{s}}{{dt}}\;=\;{f}_{{vs}}-\frac{{C}_{s}}{{\tau }_{s}},$$

where ${f}_{{vs}}$ is the carbon flux from vegetation to soil, from either litterfall, mortality or direct transfer from roots to soil.

We calculate annual ${\tau }_{v}$ (using Eq. 3), ${\tau }_{s}$ and ${f}_{{vs}}$ using model output as follows:

$${\tau }_{v}\;=\;\frac{{C}_{v}}{{{{{{{\mathrm{NPP}}}}}}}-\varDelta {C}_{v}},$$

$${\tau }_{s}\;=\;\frac{{C}_{s}}{{R}_{h}},$$

$${f}_{{vs}}\;=\;\varDelta {C}_{s}+{R}_{h},$$

where $\varDelta {C}_{v}$ and $\varDelta {C}_{s}$ are the annual changes in vegetation and soil carbon. We want to be able to define $\varDelta C$ in terms of input fluxes and turnover times only, as these are the two aggregated mechanisms driving changes in the pools. To do this, we use the fact that the pools and the input and output fluxes are generally much larger than the change in carbon pools each year to make the assumption that $\varDelta C\simeq 0$. Rearranging Eqs. 3 and 4, we can estimate the steady-state carbon pools in any particular year as:

5 $${C}_{v}\simeq {\hat{C}}_{v}\;=\;{{{{{{\mathrm{NPP}}}}}}}{\tau }_{v}$$

6 $${C}_{s}\simeq {\hat{C}}_{s}\;=\;{f}_{{vs}}{\tau }_{s},$$

where ${\hat{C}}_{v}$ and ${\hat{C}}_{s}$ are steady-state approximations of the actual carbon pools ${C}_{v}$ and ${C}_{s}$. We can now define the change in carbon pools over our 60-year study period, 1959–2020, in terms of inputs and turnover time:

7 $$\varDelta {C}_{v}\simeq \varDelta {\hat{C}}_{v}\;=\;{{{{{{\mathrm{NP}}}}}}}{{{{{{\mathrm{P}}}}}}}_{2020}{\tau }_{v,2020}-{{{{{{\mathrm{NP}}}}}}}{{{{{{\mathrm{P}}}}}}}_{1959}{\tau }_{v,1959}$$

8 $$\varDelta {C}_{s}\simeq \varDelta {\hat{C}}_{s}\;=\;{f}_{{vs},2020}{\tau }_{s,2020}-{f}_{{vs},1959}{\tau }_{s,1959}.$$

In order to separate the impacts of changes in inputs and turnover times, we next define ${{{{{{\mathrm{NP}}}}}}}{{{{{{\mathrm{P}}}}}}}_{2020}={{{{{{\mathrm{NP}}}}}}}{{{{{{\mathrm{P}}}}}}}_{1959}+\varDelta {{{{{{\mathrm{NPP}}}}}}}$, ${f}_{{vs},2020}={f}_{{vs},1959}+\varDelta {f}_{{vs}}$, and ${\tau }_{2020}={\tau }_{1959}+\varDelta \tau$. We now substitute these into Eqs. 7 and 8:

9 $$\varDelta {\hat{C}}_{v}\;=\;\varDelta {{{{{{\mathrm{NPP}}}}}}}{\tau }_{v,1959}+{{{{{{\mathrm{NP}}}}}}}{{{{{{\mathrm{P}}}}}}}_{1959}\varDelta {\tau }_{v}+\varDelta {{{{{{\mathrm{NPP}}}}}}}\varDelta {\tau }_{v}$$

10 $$\varDelta {\hat{C}}_{s}\;=\;\varDelta {f}_{{vs}}{\tau }_{s,1959}+{f}_{{vs},1959}\varDelta {\tau }_{s}+\varDelta {f}_{{vs}}\varDelta {\tau }_{s}$$

The right-hand side of the equation contains three terms corresponding to the change in carbon storage due to changes in inputs (NPP for vegetation and litterfall for soil), outputs (bulk turnover), and an interaction term. Our approach, therefore, extends the factor separation approach by ref. 21, which applied it to attribute simulated soil carbon changes into input-driven, turnover-driven change and a synergy term to also cover vegetation carbon changes.

The change in vegetation carbon due to each term can be written as: $\varDelta {\hat{C}}_{v,{{{{{{\mathrm{input}}}}}}}}=\varDelta {{{{{{\mathrm{NPP}}}}}}}{\tau }_{v,1959}$, $\varDelta {\hat{C}}_{v,{{{{{{\mathrm{output}}}}}}}}={{{{{{\mathrm{NP}}}}}}}{{{{{{\mathrm{P}}}}}}}_{1959}\varDelta {\tau }_{v}$, $\varDelta {\hat{C}}_{v,{{{{{{\mathrm{interaction}}}}}}}}=\varDelta {{{{{{\mathrm{NPP}}}}}}}\varDelta \tau$ and $\varDelta {\hat{C}}_{v}=\varDelta {\hat{C}}_{v,{{{{{{\mathrm{input}}}}}}}}$ + $\varDelta {\hat{C}}_{v,{{{{{{\mathrm{output}}}}}}}}$ + $\varDelta {\hat{C}}_{v,{{{{{{\mathrm{interaction}}}}}}}}$, with equivalent terms for soil carbon.

Finally, we adjust the one-pool steady-state estimates for the difference in the model simulations that arise due to the combination of steady-state assumption (Eqs. 5–8), the grouping of all pools into single vegetation and soil pools (as the distribution between component pools changes over time), and the linearisation not capturing the full dynamics of land carbon cycling (Eqs. 9 and 10). Therefore, the estimates of changes in carbon pools ($\varDelta {\hat{C}}_{v}$ and $\varDelta {\hat{C}}_{s}$) do not equal the actual change in C pools ($\varDelta {C}_{v}$ and $\varDelta {C}_{s}$), which are a direct model output (see Supplementary Fig. 9).

To address this issue, we calculate for each model the difference between the actual change in carbon stocks and our approximation (${\delta }_{v}=\varDelta {C}_{v}-\varDelta {\hat{C}}_{v}$ and ${\delta }_{s}=\varDelta {C}_{s}-\varDelta {\hat{C}}_{s}$). We make the simple assumption that productivity and turnover are proportionally responsible for the mismatch between steady state and the actual carbon stock, and so adjust each term on the right-hand side of Eqs. 9 and 10 by the relative magnitude of each term multiplied by ${\delta }_{v}$ or ${\delta }_{s}$ for vegetation and soil, respectively. For example, $\varDelta {\hat{C}}_{v,{{{{{{\mathrm{input}}}}}}}}$ is adjusted by adding:

11 $$\frac{{{{{{\rm{|}}}}}}\varDelta {\hat{C}}_{v,{{{{{{\mathrm{input}}}}}}}}{{{{{\rm{|}}}}}}}{{{{{{\rm{|}}}}}}\varDelta {\hat{C}}_{v,{{{{{{\mathrm{input}}}}}}}}{{{{{\rm{|}}}}}}+{{{{{\rm{|}}}}}}\varDelta {\hat{C}}_{v,{{{{{{\mathrm{output}}}}}}}}{{{{{\rm{|}}}}}}+{{{{{\rm{|}}}}}}\varDelta {\hat{C}}_{v,{{{{{{\mathrm{interaction}}}}}}}}{{{{{\rm{|}}}}}}}*{\delta }_{v}.$$

We calculate Eqs. 9 and 10 for each of the eighteen models, apply the appropriate scaling and use them to produce Fig. 4.

### Uncertainty analysis

The spread of model estimates of changes in carbon stocks and fluxes is used to determine the uncertainty of our results. For land flux estimates (NBP) and $\varDelta {C}_{v}$ and $\varDelta {C}_{s}$, we show ‘mean ± std. dev.’. We decompose the uncertainty (defined as the model spread) in $\varDelta {\hat{C}}_{v}$ and $\varDelta {\hat{C}}_{s}$ into four components: baseline input (${{{{{{\mathrm{NP}}}}}}}{{{{{{\mathrm{P}}}}}}}_{1959}$ or ${f}_{{vs},1959}$), change in inputs ($\varDelta {{{{{{\mathrm{NPP}}}}}}}$ or $\varDelta {f}_{{vs}}$), baseline turnover (${\tau }_{v,1959}$ or ${\tau }_{s,1959}$), and change in turnover ($\varDelta {\tau }_{v}$ or $\varDelta {\tau }_{s}$). For each of these terms in Eqs. 9 and 10, we calculate the standard deviation in $\varDelta {\hat{C}}_{v}$ and $\varDelta {\hat{C}}_{s}$using the multi-model mean values of all other terms in the equations and the individual model values for that term.

## Supplementary information

Supplementary Information File

## Data Availability

All code and post-processed data generated in this study are available at 10.5281/zenodo.6884342. The raw model output is available at the following sftp site: trendy-v10@trendy.ex.ac.uk. Access will be granted by first contacting Stephen Sitch (S.A.Sitch@exeter.ac.uk).
