# Supplementary material for: Process-oriented analysis of dominant sources of uncertainty in the land carbon sink
Source: Nat Commun. 2022 Aug 15;13:4781. doi: 10.1038/s41467-022-32416-8 (PMC9378641; doi:10.1038/s41467-022-32416-8)
Supplement: Supplementary file 1 — Supplementary Information File [file 41467_2022_32416_MOESM1_ESM.pdf]

Supplementary Information File

Process-oriented analysis of dominant sources of uncertainty in the land carbon sink

Contents:

Figures S1-S9

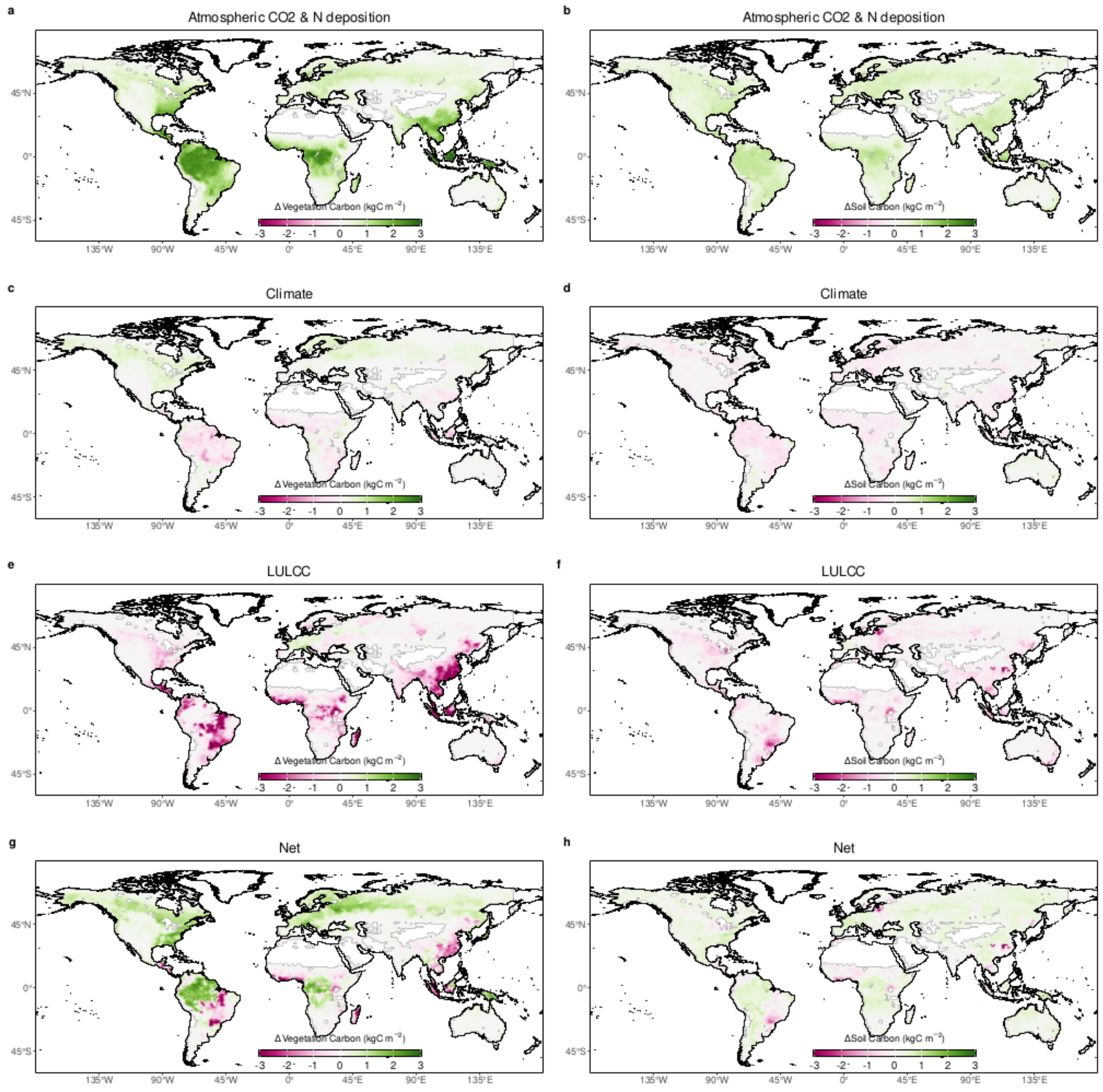

Supplementary Figure 1 – Spatial change in carbon stocks from 1959 to 2020
Maps show the DGVM mean change in (**a,c,e,g**) vegetation and (**b,d,f,h**) soil carbon (kgC m^{-2}), due to (**a,b**) rising atmospheric CO2 and N deposition, (**c,d**) changes in climate, (**e,f**) LULCC, and (**g,h**) net change with all drivers varying.

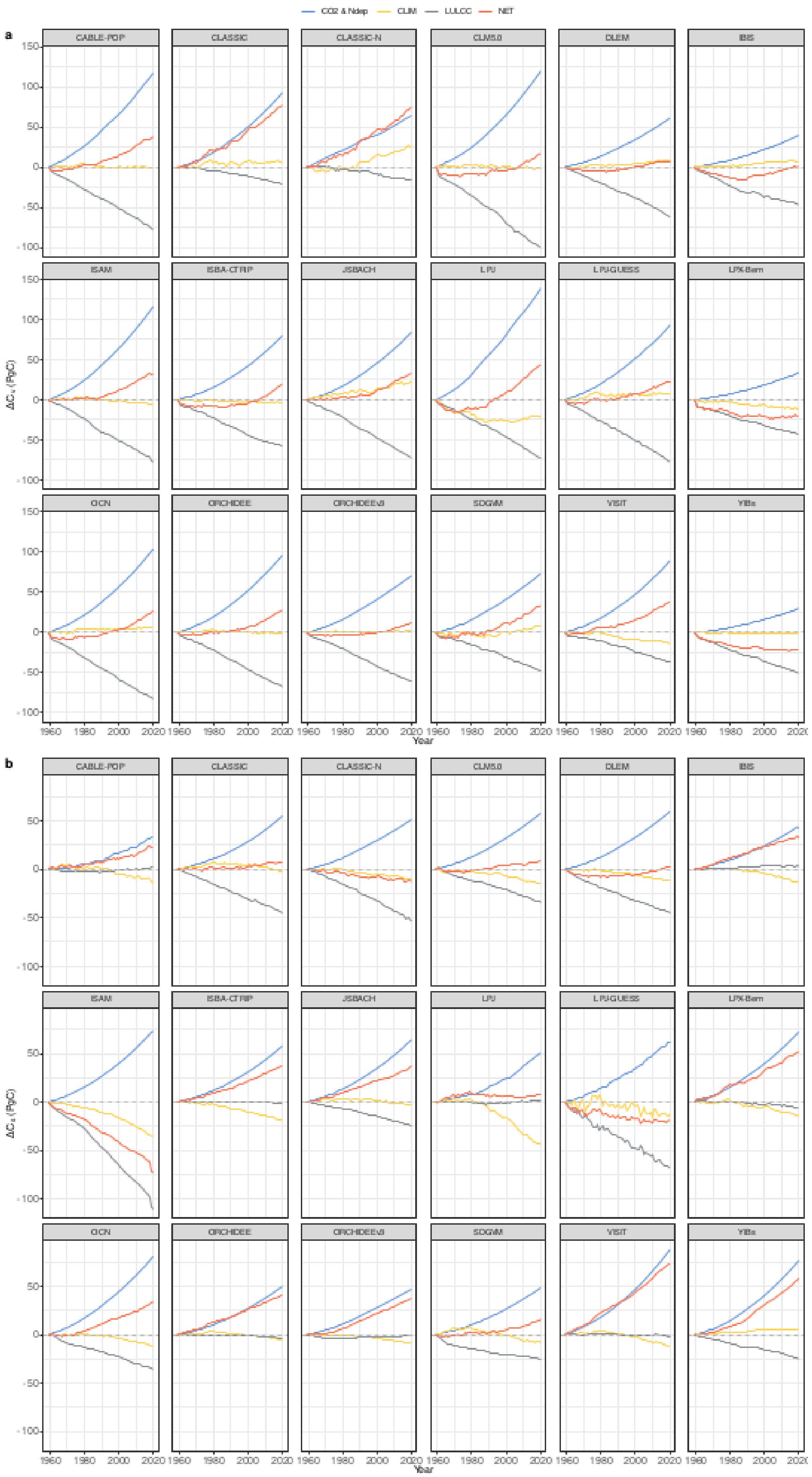

Supplementary Figure 2 – Change in global carbon stocks for individual models from 1959 to 2020

Temporal change in global (**a**) vegetation (C_v) and (**b**) soil (C_s) carbon stocks over 1959-2020 (PgC) due to CO2 and N deposition (blue), climate (yellow), LULCC (grey), and net changes (red) for each DGVM.

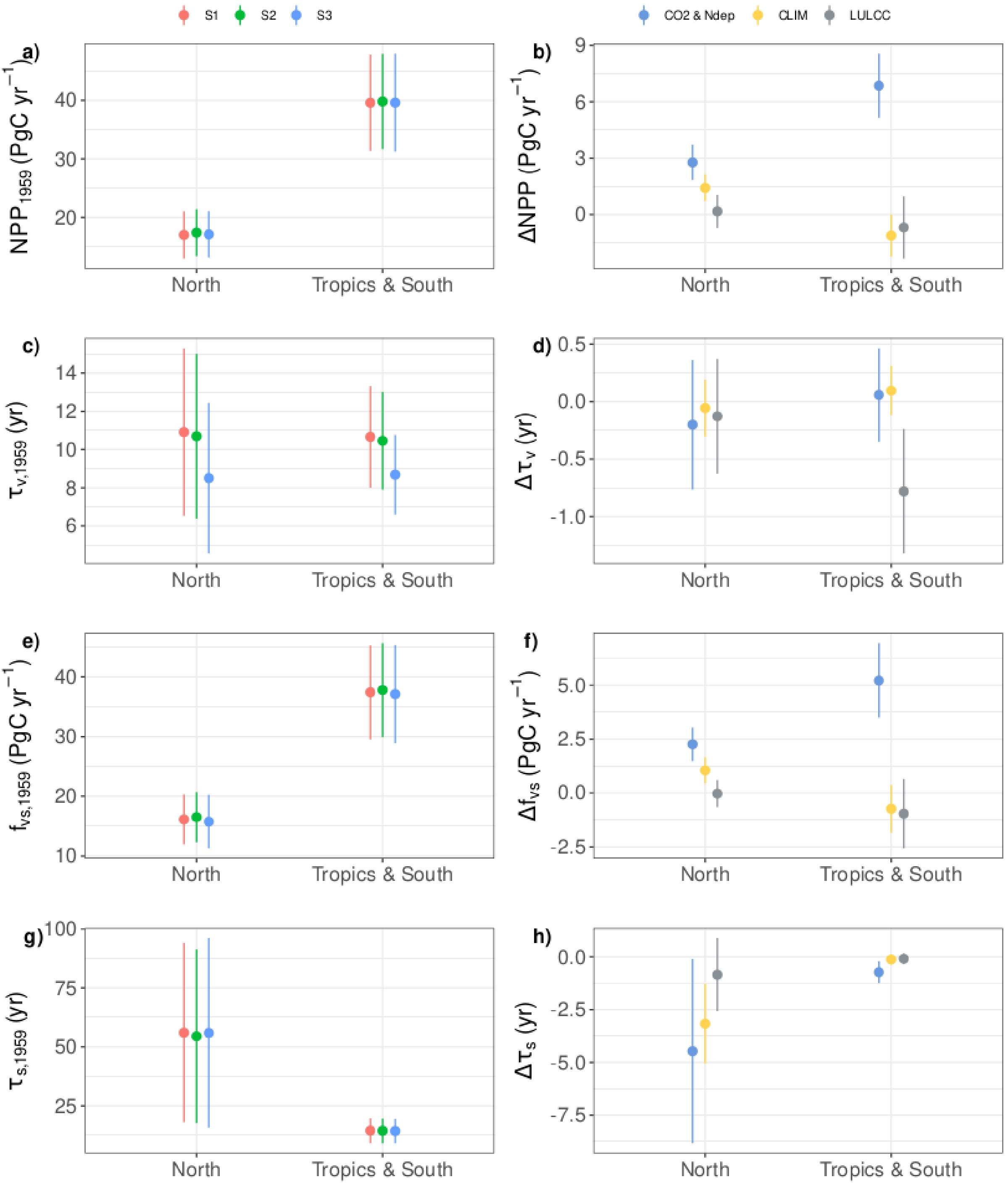

Supplementary Figure 3 – Summary of regional carbon fluxes and turnover times. (**a,c,e,g**) Baseline (in the year 1959) and (**b,d,f,h**) changes (over 1959-2020) in regional (**a,b**) NPP (PgC yr^{-1}), (**c,d**) vegetation turnover, τ_v (yr), (**e,f**) soil carbon inputs from vegetation, f_{vs} (PgC yr^{-1}), and (**g,h**) soil turnover, τ_s (yr). Dots and lines represent the DGVM mean ± 1σ. For the baseline values, output for the 3 runs (S1-S3, see methods in main ms for details on run descriptions) is shown. Changes in values over time are attributed to CO2 and N deposition (blue), climate (yellow), and LULCC (grey).

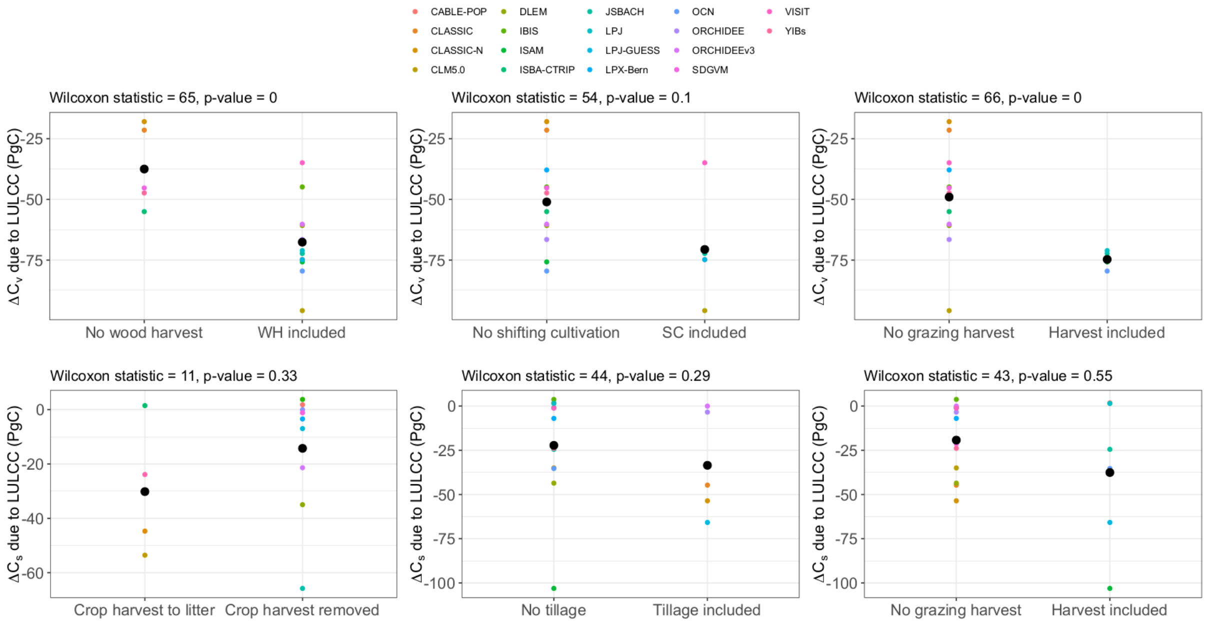

Supplementary Figure 4 – Change in global carbon stocks due to LULCC depend on included processes

The change in (top row) vegetation and (bottom row) soil carbon (PgC) due to LULCC for each DGVM, with the models grouped by process representation. Large black dots show the model mean for each group. Wilcoxon statistic and associated p-value is shown for each LULCC process. P-values < 0.1 show a significant (10% level) difference between the two groups and provides an indication whether the inclusion of a certain process leads to a systematic difference between models in simulated changes in carbon stocks.

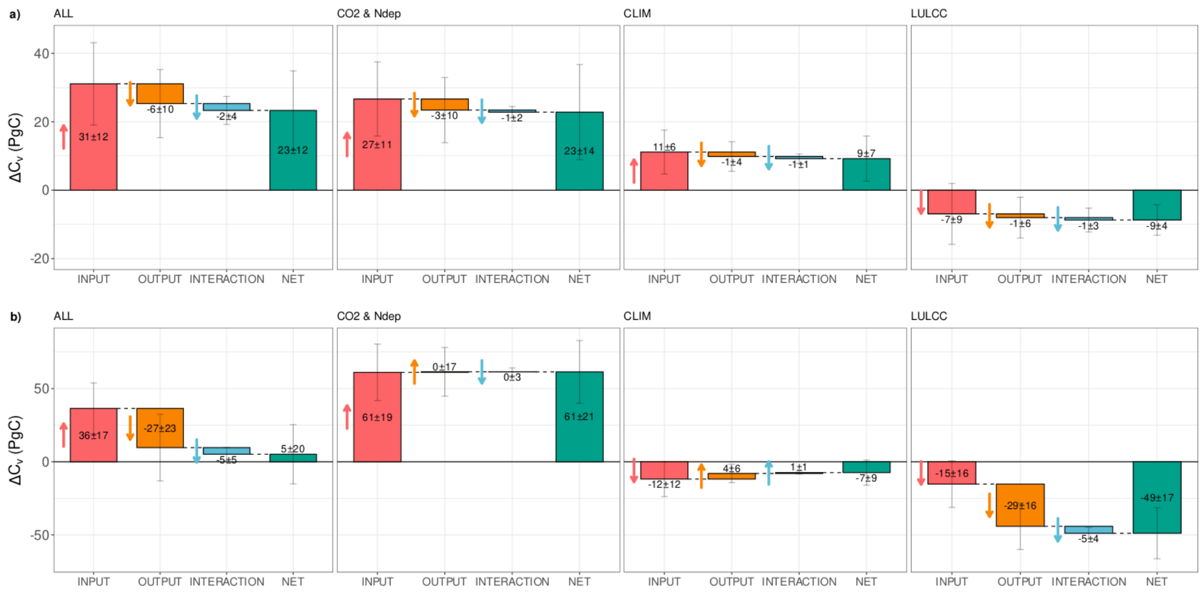

Supplementary Figure 5 - Process and driver attribution of changes in regional vegetation carbon stocks.

Change in (**a**) northern (>30°N) and (**b**) tropical and southern (<30°N) vegetation (ΔC_v) carbon stocks over 1959-2020 (PgC). The contribution to net (green bars) changes in carbon stocks from changes in NPP ($\Delta NPP\tau_{v,1959}$, red bars), vegetation turnover ($NPP_{1959}\Delta\tau_v$, orange bars), and the interaction term ($\Delta NPP\Delta\tau_v$, blue bars) are shown. The bars depict the multi-model mean with the range as ±1σ of the DGVMs. The arrows show the direction of change in carbon stocks due to each process.

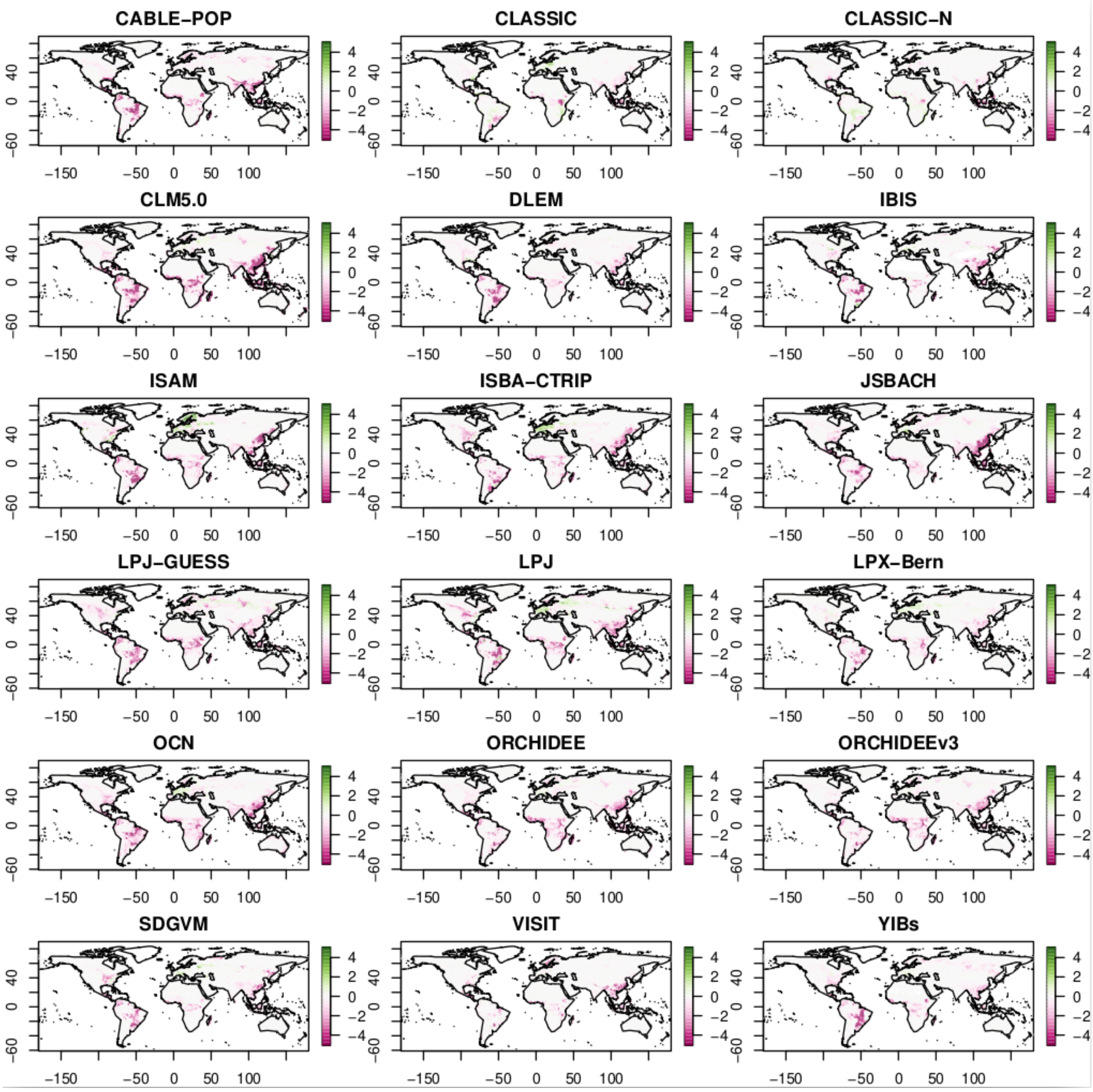

Supplementary Figure 6 – Impact of LULCC on vegetation carbon

Maps show individual model changes in biomass (kgC m^{-2}) from 1959 to 2020 due to LULCC, calculated as the difference between S2 and S3 simulations. Positive (green) values indicate a gain of carbon, whereas negative (pink) values indicate losses of carbon.

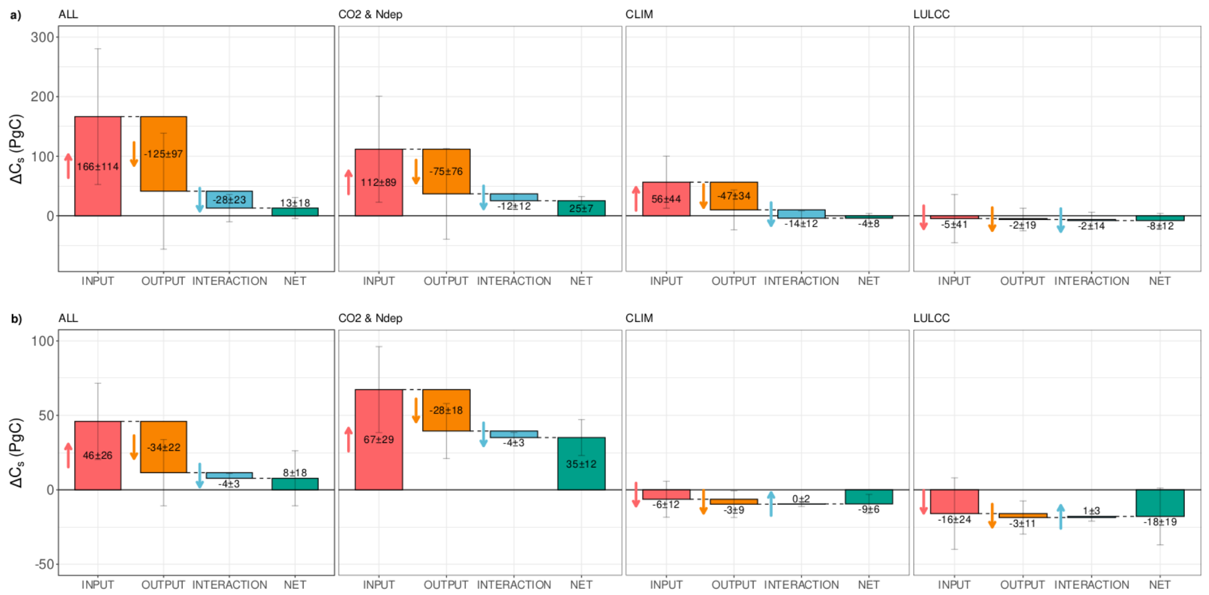

Supplementary Figure 7 - Process and driver attribution of changes in regional soil carbon stocks.

Change in (**a**) northern (>30°N) and (**b**) tropical and southern (<30°N) soil (ΔC_s) carbon stocks over 1959-2020 (PgC). The contribution to net (green bars) changes in carbon stocks from changes in vegetation to soil flux ($\Delta f_{vs}\tau_{s,1959}$, red bars), soil turnover ($f_{vs,1959}\Delta\tau_s$, orange bars), and the interaction term ($\Delta f_{vs}\Delta\tau_s$, blue bars) are shown. The bars depict the multi-model mean with the range as ±1σ of the DGVMs. The arrows show the direction of change in carbon stocks due to each process.

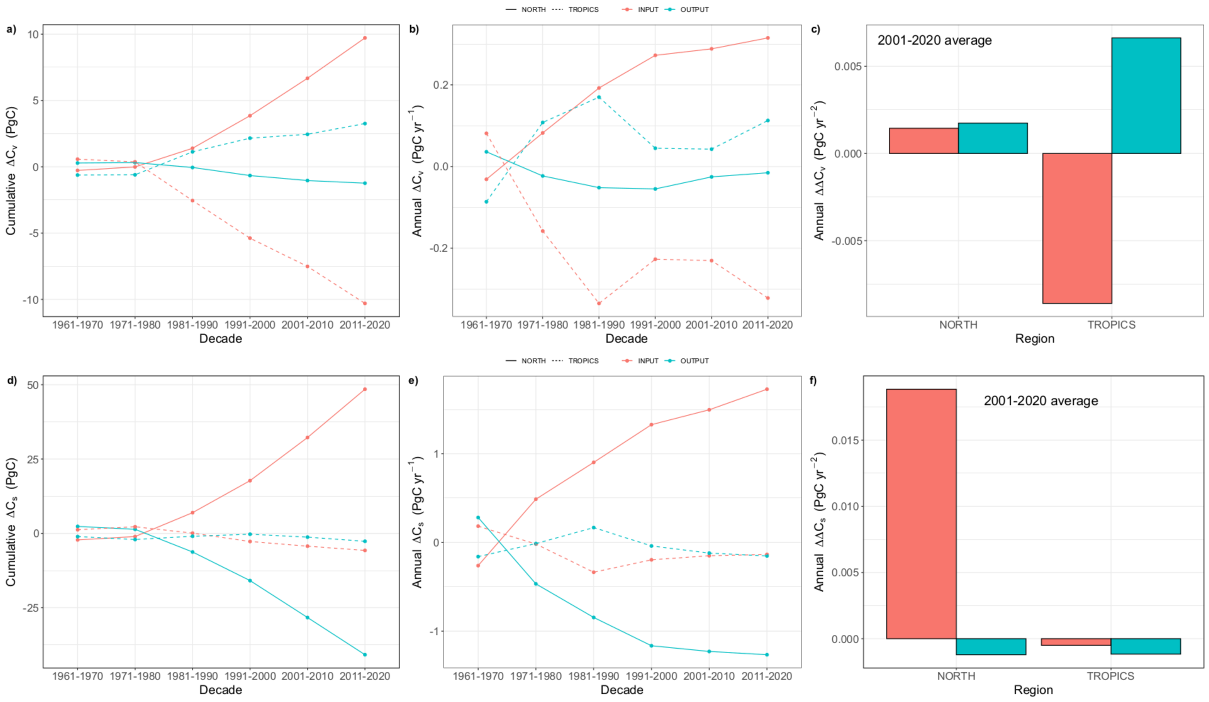

Supplementary Figure 8 - Acceleration of climate impacts on carbon cycling
(**a,b**) Cumulative (PgC) and (**b,e**) annual (PgC yr^{-1}) changes in (**a,b**) vegetation and (**d,e**) soil carbon stocks due to changes in climate in northern (solid lines) and tropical/southern (dashed lines) latitudes. Changes in carbon stocks are decomposed (using equations 9, 10 in Methods) into changes driven by inputs (NPP for vegetation, litterfall for soil) and outputs (turnover), in red and blue, respectively. (**c,f**) The acceleration of carbon stock changes is calculated as the difference from one year to the next in annual changes in stocks. Here we depict the mean acceleration (PgC yr^{-2}) over 2011-2020 for (**c**) vegetation and (**f**) soil.

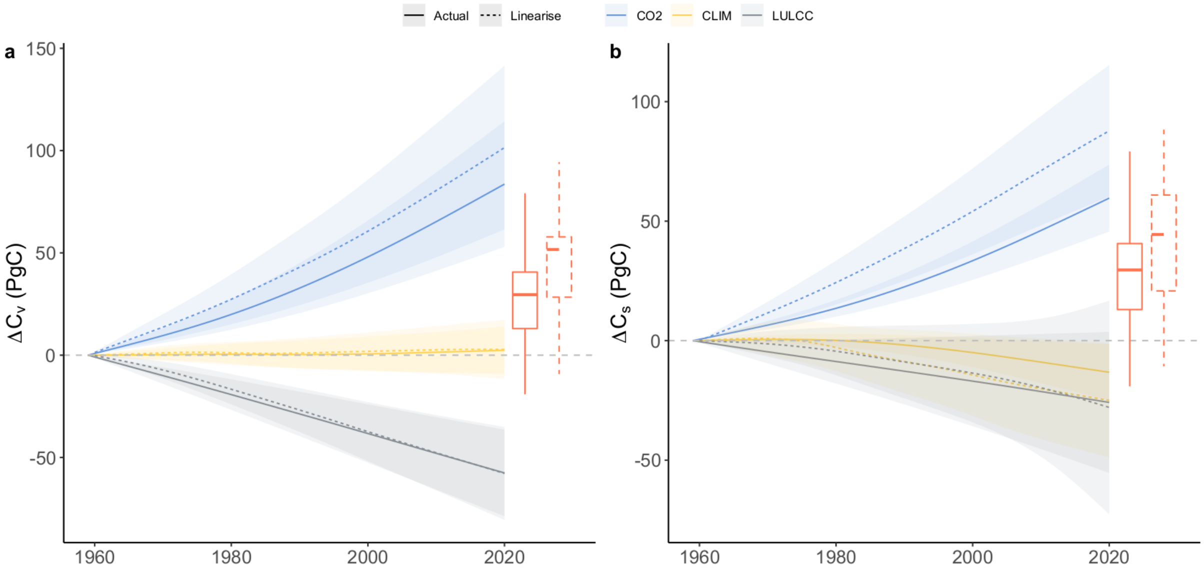

Supplementary Figure 9 – Linearised changes in carbon stocks do not match actual simulated changes

Changes in global (**a**) vegetation and (**b**) soil carbon stocks over 1959-2020 due to CO_2 and N deposition (blue), climate (yellow), LULCC (grey), with the net change by 2020 shown with a boxplot. Solid lines show the direct model output and dashed lines the linearised estimate (see equations 9 & 10 in the main ms). The difference between the solid and dashed lines prompted the need to scale the terms in equations 9 & 10 to match the actual modelled changes (see methods in main ms).
